# *Hox* Paralog Group 2 Genes Control the Migration of Mouse Pontine Neurons through Slit-Robo Signaling

**DOI:** 10.1371/journal.pbio.0060142

**Published:** 2008-06-10

**Authors:** Marc J Geisen, Thomas Di Meglio, Massimo Pasqualetti, Sebastien Ducret, Jean-François Brunet, Alain Chedotal, Filippo M Rijli

**Affiliations:** 1 Institut de Génétique et de Biologie Moléculaire et Cellulaire, CNRS/INSERM/ULP, UMR 7104, CU de Strasbourg, Illkirch, France; 2 CNRS UMR 7102 Université Pierre et Marie Curie–Paris 6, Paris, France; 3 Friedrich Miescher Institute, Basel, Switzerland; 4 CNRS UMR8542, Ecole Normale Supérieure, Paris, France; Baylor College of Medicine, United States of America

## Abstract

The pontine neurons (PN) represent a major source of mossy fiber projections to the cerebellum. During mouse hindbrain development, PN migrate tangentially and sequentially along both the anteroposterior (AP) and dorsoventral (DV) axes. Unlike DV migration, which is controlled by the Netrin-1/Dcc attractive pathway, little is known about the molecular mechanisms guiding PN migration along the AP axis. Here, we show that Hoxa2 and Hoxb2 are required both intrinsically and extrinsically to maintain normal AP migration of subsets of PN, by preventing their premature ventral attraction towards the midline. Moreover, the migration defects observed in *Hoxa2* and *Hoxb2* mutant mice were phenocopied in compound *Robo1;Robo2*, *Slit1;Slit2*, and *Robo2;Slit2* knockout animals, indicating that these guidance molecules act downstream of *Hox* genes to control PN migration. Indeed, using chromatin immunoprecipitation assays, we further demonstrated that Robo2 is a direct target of Hoxa2 in vivo and that maintenance of high Robo and Slit expression levels was impaired in *Hoxa2* mutant mice. Lastly, the analysis of *Phox2b*-deficient mice indicated that the facial motor nucleus is a major Slit signaling source required to prevent premature ventral migration of PN. These findings provide novel insights into the molecular control of neuronal migration from transcription factor to regulation of guidance receptor and ligand expression. Specifically, they address the question of how exposure to multiple guidance cues along the AP and DV axes is regulated at the transcriptional level and in turn translated into stereotyped migratory responses during tangential migration of neurons in the developing mammalian brain.

## Author Summary

In the developing central nervous system, neurons migrate sometimes over long distances from their birthplace to their final location, where they condense in specific nuclei. The precise positioning of migrating neurons is critical to the building of ordered connectivity with their target partners. Little is known about how exposure of migrating neurons to simultaneous attractive and repulsive guidance cues may be integrated at the transcriptional level and in turn translated into directional migratory responses specific for each neuronal population. Here, we focus on the molecular mechanisms regulating the directionality of long-distance migration of pontine neurons in the mouse brainstem. Such neurons belong to the so-called precerebellar system, which is essential for coordinated motor activity, and provide the principal input to the cerebellum. We provide evidence for the implication of homeodomain transcription factors of the *Hox* gene family in the control of pontine neuron migration along the brain rostrocaudal axis. We identify the guidance receptor *Robo2* as a direct target gene of the *Hoxa2* gene. We further show that repulsive signaling mediated through the Robo2 receptor expressed in migrating neurons and its ligand Slit2 secreted from the facial motor nucleus are key components of the molecular guidance system that maintains caudorostral migration and prevents premature attraction towards the brainstem ventral midline. Our data provide a conceptual framework to understand how transcriptional regulation of the response to environmental guidance cues controls stereotyped neuronal migratory behavior in the developing mammalian brain.

## Abstract 3

Homeodomain-containing Hox factors are well-known for controlling body patterning, but a new study reveals a novel role for these genes in controlling the long-distance tangential migration of neurons in the mouse brain stem through transcriptional regulation of Robo receptor-Slit ligand family guidance molecules.

## Introduction

In the developing central nervous system (CNS), neurons migrate sometimes over long distances from their birthplace to their final location, where they condense in specific nuclei. As neuronal function depends upon precise connectivity with their targets, the final positioning of migrating neurons is critical to the building of ordered connectivity between pre- and postsynaptic partners. In many brain regions, neurons migrate in a tangential direction, orthogonal to the radial axis and independently of radial glia, resulting in mixing of cells that originated from distinct ventricular regions [1]. Mounting evidence suggests that tangentially migrating neurons are guided during their journey by the same set of attractive and repulsive guidance cues that regulate axonal pathfinding and topographical mapping [2]. However, little is known about how exposure of migrating neurons to several simultaneous extrinsic inputs along the orthogonal axes of the brain may be integrated at the transcriptional level and in turn translated into directional migratory responses specific for each neuronal population.

In the mouse hindbrain, the precerebellar system is a suitable model to study the molecular mechanisms controlling long-distance tangential migration. Precerebellar nuclei are essential for coordinated motor activity and provide the principal input to the cerebellum [3]. They convey information to the cerebellum through the climbing and mossy fiber projection systems. Although the only source of climbing fibers is the inferior olivary nucleus (ION), mossy fibers have multiple origins, such as the lateral reticular (LRN) and external cuneate (ECN) nuclei in the posterior hindbrain, and the pontine gray (PGN) and reticulotegmental (RTN) nuclei, collectively referred thereafter as pontine neurons (PN), in the anteroventral hindbrain [4].

The early patterning of the mouse hindbrain along the anteroposterior (AP) axis is characterized by a segmentation process into distinct morphological segments called rhombomeres, resulting in spatial segregation of the neuroepithelium contributing to each segment [5]. Distinct precerebellar neuronal populations are contributed by rhombomeric portions of the rhombic lip, a stripe of neuroepithelium that arises dorsally at the interface with the roof plate and runs throughout the rostrocaudal extent of the hindbrain, giving rise to spatiotemporally defined sequences of migratory populations [4,6–12]. Indeed, rhombomere (r)1 rhombic lip derivatives specifically contribute to the external granular layer (EGL) of the cerebellum [12], whereas a recent fate mapping study subdivided the mouse r2–r8 rhombic lip into two distinct domains: the auditory lip, extending from r2 to r5 and giving rise to the brainstem cochlear nuclear complex; and the precerebellar lip, which generates the precerebellar nuclei, running posteriorly to r5 [6]. Mossy fiber projection neurons migrate along two distinct subpial streams: the posterior extramural stream, whose neurons contribute to the LRN and the ECN, and the anterior extramural stream that is formed by the PN contributing to the PGN and the RTN [4]. The latter undergo a long-distance rostral migration through several rhombomeric domains before turning ventrally to reach a stereotypic anteroventral position in the mature brain stem. The ventral pathway of PN migration was shown to involve Deleted in colon cancer (Dcc)/Netrin-1–mediated chemoattraction [13–15] and the Slit receptor Robo3 [16].

Here, we studied the intrinsic and extrinsic molecular mechanisms regulating the directionality of migration of PN along the rostral pathway. During embryo development, the transcriptional readout of AP positional information is provided by the *Hox* gene family of transcription factors [5]. In the developing hindbrain, *Hox* genes have been involved in providing segmental identity to rhombomeres and rostrocaudal patterning information to developing neurons [5,17]. More recently, it has been shown that *Hox* gene expression is maintained up to late stages of development in specific neuronal subpopulations in the hindbrain and spinal cord, where they may be important for the establishment of topographically organized sensory and motor circuits [18–20]. However, the potential role of *Hox* genes in orienting directional neuronal migration through regulation of guidance molecules remain largely unknown.

Herein, we found that the paralog group (PG)2 *Hox* genes, *Hoxa2* and *Hoxb2*, are required to maintain normal migration of PN along the rostral pathway. In *Hoxa2* and *Hoxb2* mutants, subsets of PN prematurely migrated ventrally, settling at ectopic posterior locations. Interestingly, the PN migratory defects observed in PG2 *Hox* mutants phenocopied those found in *Robo1;Robo2*, *Slit1;Slit2*, and *Robo2;Slit2* compound mutants. Furthermore, in PG2 *Hox* mutants the expression levels of *Robo2* and *Slit2* were decreased and chromatin immunoprecipitation assays demonstrated the direct binding of Hoxa2 on the *Robo2* locus. In addition, we identified the facial motor nucleus (FMN), which is located in ventrolateral r6, as an important source of Slit ligands for Robo receptor–expressing PN. This is supported by the finding that in the *Phox2b* knockout mice, which completely lack the FMN, the PN undergo the same ectopic and premature ventral migratory defects as observed in *Hox* PG2, *Robo1;Robo2*, *Slit1;Slit2*, or *Robo2;Slit2* compound mutants. Altogether, our data provide important novel insights into the intrinsic and extrinsic molecular determinants involved in tangential migration of PN neurons along the AP axis.

## Results

### Rhombomeric Mapping of Migration Pathway and *Hox* Gene Expression of Pontine Neurons

**Figure 1:**
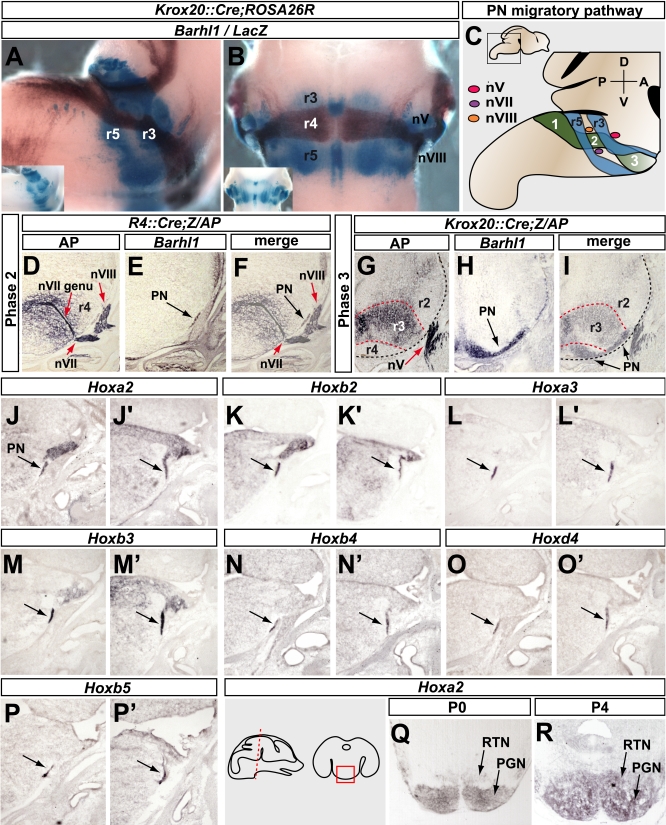
Rhombomeric Mapping of Migration Pathway and *Hox* Gene Expression of Pontine Neurons (A–C) Lateral (A) and ventral (B) views of E15.5 *Krox20::Cre;ROSA26R* whole-mount brain*.* The progenies of r3 and r5 are traced through lacZ activity. Subsequently, the brain was hybridized with a *Barhl1* antisense probe. Insets in (A) and (B) show whole-mount brains after staining for lacZ activity and before in situ hybridization with Barhl1. *Barhl1^+^* neurons stream from a dorsoposterior to an anteroventral location, crossing the r5- and r4-derived territory before entering the r3-derived domain where they turn to migrate ventrally. In (B), PN finally settle in the r4-derived territory, just abutting r3- and r5-derived domains. The migration is subdivided into three distinct phases (1–3) as indicated in the summary diagram in (C). (D–F) Adjacent coronal cryostat sections of E15.5 *R4::Cre;Z/AP* brain at the level of the rostral phase (phase 2) of PN migration, stained for alkaline phosphatase activity (D) or hybridized with a *Barhl1* probe (E). (F) Merge of (D) and (E). nVII and nVIII (arrows) exit the brain at the level of the r4-derived domain (D). During phase 2, PN migrate in a corridor beneath the nVIII and above the nVII (F). (G and H) Adjacent coronal cryostat sections of E15.5 *Krox20::Cre;Z/AP* brain at the level of the final ventral phase (phase 3) of PN migration stained for alkaline phosphatase activity (AP) (G) or hybridized with a *Barhl1* probe (H). The black dashed line in (G) delimits the outline of the brain, the red dashed lines delimit the r3-derived territory. (I) Merge of (G) and (H). PN neurons migrate through the r3-derived territory before they reenter and settle in the r4-derived domain ventrally (I). PN never enter the r2-derived territory (I). The black dashed line in (I) delimits the outline of the brain; the red dashed lines delimit the r3-derived territory. Note that the nV exits the brain at the level of the r2-derived domain (G and I). (J–P') E14.5 cryostat hindbrain sections hybridized with *Hoxa2* (J and J'), *Hoxb2* (K and K'), *Hoxa3* (L and L'), *Hoxb3* (M and M'), *Hoxb4* (N and N'), *Hoxd4* (O and O'), and *Hoxb5* (P and P') probes. The arrows show expression in migrating PN. (J–P) correspond to sections at the level of nVII (phase 2), only the right side of the section is shown. (J′–P′) correspond to sections posterior to nVII (phase 1). (Q and R) Cryostat sections of P0 (Q), and P4 (R) brains hybridized with a *Hoxa2* probe. The section level is indicated by the red box in the left diagram. Arrows show *Hoxa2* expression in the PGN and RTN nuclei. nV, trigeminal nerve; nVII, facial nerve; nVIII, vestibulocochlear nerve.

Tangentially migrating PN originate in the r6–r8 rhombic lip between embryonic day (E)13.5 and E17.5 [10]. They migrate first ventrally and then rostrally until they reach a final anteroventral position in the pontine primordium (Figure 1A–1C; [6]). During their migration, PN navigate across distinct rhombomere-derived territories and express several molecular markers, including the homeobox-containing gene *Barhl1* (Figure 1A, 1B, 1E, and 1H; [21]). To map the migratory route of PN in relationship to rhombomere territories, we simultaneously performed lacZ staining and in situ hybridization with *Barhl1* on whole-mount brains or tissue sections from *Krox20::Cre;ROSA26R* (Figure 1A and 1B), *Krox20::Cre;Z/AP* (Figure 1G–1I), or *R4::Cre;Z/AP* (Figure 1D–1F) mouse lines [20,22–24] in which lacZ or alkaline phosphatase expression is permanently activated in r3 and r5 or r4 progenies, respectively. The migratory pathway of *Barhl1^+^* PN neurons can be divided into three distinct phases (Figure 1A–1C). First, PN undertake a short ventral migration upon leaving the r6–r8 rhombic lip (phase 1). In the next step (phase 2), PN turn rostrally, parallel to the AP axis. They travel through r5 and r4, where they pass between the vestibulocochlear (VIIIth, dorsally) and the facial (VIIth, ventrally) nerve roots (Figure 1C–1F; [25]) and continue migrating through r3 until they reach the trigeminal (Vth) nerve root located in the caudal aspect of r2 (Figure 1A–1C). Interestingly, the PN stream never enters r2 but makes an abrupt change of direction and undertakes a final ventral migration within r3 (phase 3) (Figure 1A–1C and 1G–1I). In the latter aspect of their migration, PN leave r3, reenter r4, and fan out within the r4-derived domain until they finally settle near the floor plate, abutting the r3- and r5-derived territories (Figure 1B and 1G–1I; [6]).

Such a stereotyped migratory pathway suggested that, in addition to dorsoventral (DV) positional cues, PN may express and/or respond to molecular cues along the AP axis, such as the *Hox* gene products. Indeed, in situ hybridization analysis using specific *Hox* antisense probes revealed that migrating PN expressed a *Hox* program characteristic of an r6–r8 axial origin, namely *Hox* PG2–5 genes. Specifically, transcripts for *Hoxa2*, *Hoxb2*, *Hoxa3*, *Hoxb3*, *Hoxb4*, *Hoxd4*, and *Hoxb5* were expressed in PN throughout their migration and in the settling pontine nuclei (Figure 1J–1R; and unpublished data). Such expression patterns suggested that PN may be endowed with molecular information as to their origin and relative position along the AP axis throughout their migration.

### *Hoxa2* and *Hoxb2* Are Required for Migration of Subsets of Pontine Neurons along the Anteroposterior Axis

To address the potential involvement of *Hox* genes in PN rostral migration, we focused on the PG2 genes, *Hoxa2* and *Hoxb2*. These genes perform important roles in rostral hindbrain patterning [20,26–29], and their expression is maintained throughout migration, nucleogenesis, and establishment of connectivity to cerebellum of PN (both RTN and PGN) up to postnatal stages (Figure 1J–1K′, 1Q, and 1R; and unpublished data).

**Figure 2:**
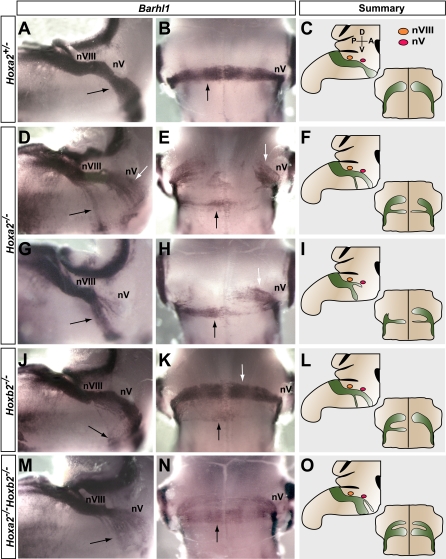
Migration Defects of Pontine Neurons in Single *Hoxa2*, *Hoxb2*, and Compound *Hoxa2;Hoxb2* Mutant Mice (A–I) Lateral (A, D, and G) and ventral (B, E, and H) views of *Hoxa2* ^+/−^ (A and B) and *Hoxa2^−/−^* (D, E, G, and H) whole-mount brains at E14.5 hybridized with a *Barhl1* probe to visualize migrating PN. In *Hoxa2^−/−^* specimen (D, E, G, and H), the black arrows show ectopic *Barhl1^+^* cells prematurely migrating towards the midline. The white arrows indicate PN migrating along the normal pathway. Note that the migration phenotype is asymmetrically and variably distributed when comparing distinct mutants or both sides of a given specimen (D, E, G, and H). (C, F, and I) Diagrams summarizing the normal (C) and abnormal (F and I) PN migratory phenotypes shown in (A and B) and (D, E, G, and H), respectively. (J–L) Lateral (J) and ventral (K) views of *Hoxb2^−/−^* mutant brain hybridized with *Barhl1*. Similar to *Hoxa2^−/−^* mutants, ectopic *Barhl1^+^* cells leave the stream of PN and prematurely migrate ventrally (black arrows). (L) Summary diagram of the *Hoxb2* deficient phenotype. (M–O) Lateral (M) and ventral (N) views of *Hoxa2^−/−^*;*Hoxb2^−/−^* double mutant brain hybridized with *Barhl1.*(O) Summary diagram of the *Hoxa2^−/−^*;*Hoxb2^−/−^* mutant phenotype. Note that *Hoxa2^−/−^*;*Hoxb2^−/−^* mutants appear to display more ectopic PN as compared to single mutants. nV, trigeminal nerve; nVIII, vestibulocochlear nerve.

In *Hoxa2* heterozygous mutants [30], PN displayed a normal migratory behavior as assessed by whole-mount in situ hybridization on E14.5 and E15.5 hindbrains with antisense probes for known markers of migrating PN, such as *Barhl1* (Figure 2A–2C; [21]), *Pax6*, and *Tag1* ([31,32]; unpublished data). In contrast, in *Hoxa2^−/−^* homozygous mutant mice, we observed defects of PN navigation along the rostral pathway (Figure 2D–2I). Although migratory abnormalities could be observed in all *Hoxa2^−/−^* mutants (*n* = 15), they were variably penetrant. In some cases (*n* = 10), small cohorts of neurons left the stream prematurely and migrated ventrally towards the midline (Figure 2D–2F). Ectopic neurons expressed *Barhl1*, *Pax6*, and *Tag1*, and often condensed in small ectopic nuclear formations close to the midline, posterior to the normal location of pontine nuclei (Figure 2D and 2E; and unpublished data). In these mutants, only a subset of PN migrated ectopically, whereas the bulk of the stream maintained a caudorostral migratory path. In order to investigate whether this subset was a random or specific subpopulation of PN, we analyzed the status of the RTN and PGN by in situ hybridization with *Barhl1* and *Pax6* antisense probes on *Hoxa2^−/−^* brains at E17.5 days postcoitum (dpc) (Figure S3, and unpublished data). A general slight reduction of both PGN and RTN was apparent (Figure S3K and S3L), suggesting that the *Hoxa2* inactivation randomly induced the ectopic migration of subsets of neurons contributing to both nuclei. In further support of this idea, in a number of mutant fetuses (*n* = 5), we observed more-severe defects in which almost the entire PN stream undertook a premature ventral migration (Figure 2G and 2H). It is also noteworthy that the abnormal migratory phenotypes were often asymmetrically distributed among the two sides (Figure 2H and 2I; and unpublished data). Finally, by in situ hybridization with *Barhl1*, *Pax6*, *Robo3*, and *Tag1* antisense probes on E13.5, E14.0, E14.5, and E17.5 whole-mount brains, we found ectopic migrations throughout development of PN, arguing that the *Hoxa2* inactivation did not selectively affect neurons migrating within a particular time window (Figures 2D–2I and S3A–S3J).

Altogether, these data indicated stochastic compensation for the loss of *Hoxa2* along the AP migratory path of PN. This may result from partial functional redundancy with other *Hox* genes expressed in migrating PN (Figure 1). Indeed, premature ventral migrations of PN could also be observed in some *Hoxb2^−/−^* (Figure 2J–2L; *n* = 2 out of 6). Moreover, *Hoxa2^−/−^;Hoxb2^−/−^* mutant specimen (*n* = 3; Figure 2M–2O) appeared to display more ectopic PN as compared to single mutants (compare Figure 2D–2L and 2M–2O), thus indicating some degree of functional redundancy and genetic interaction among *Hox* PG2 genes.

In summary, these results indicated that *Hoxa2*, and to a lesser extent *Hoxb2*, may regulate the response of subsets of PN to environmental cues to precisely maintain their rostral migratory route, thus ultimately contributing to control the final location of pontine nuclei along the AP axis.

### Autonomous and Nonautonomous Roles of *Hoxa2* in Regulating Rostral Migration of Pontine Neurons

*Hox* PG2 may be intrinsically required in PN throughout migration. Alternatively, or in addition, they may be required nonautonomously to pattern the environment through which PN migrate and to which PN respond in order to direct their rostral migration.

**Figure 3:**
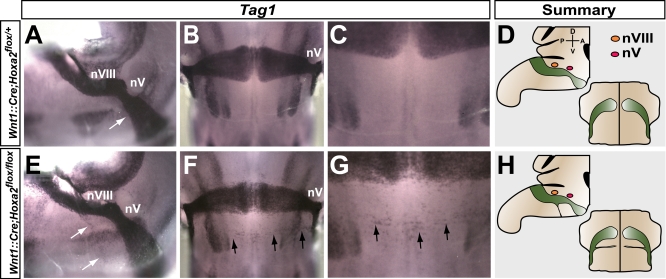
Intrinsic Requirement for *Hoxa2* in PN Migration (A–H) Conditional inactivation of *Hoxa2* in *Wnt1^+^* rhombic lip-derived neurons. Lateral (A and E) and ventral (B, C, F, and G) views of E15.5 *Wnt1::Cre;Hoxa2^flox/+^* heterozygous (A–C) and *Wnt1::Cre;Hoxa2^flox/flox^* homozygous (E–G) mutant whole-mount brains hybridized with a *Tag1* probe. In homozygous mutants (E–G), ectopic *Tag1^+^* cells appear to prematurely migrate towards the midline (arrows in [E and F]). (C and G) Higher magnifications of ventral views shown in (B and F), respectively. (D and H) Summary of the PN migratory phenotypes in *Wnt1::Cre;Hoxa2^flox/+^* (D) and *Wnt1::Cre;Hoxa2^flox/flox^* (H) brains. nV, trigeminal nerve; nVIII, vestibulocochlear nerve.

**Figure 4:**
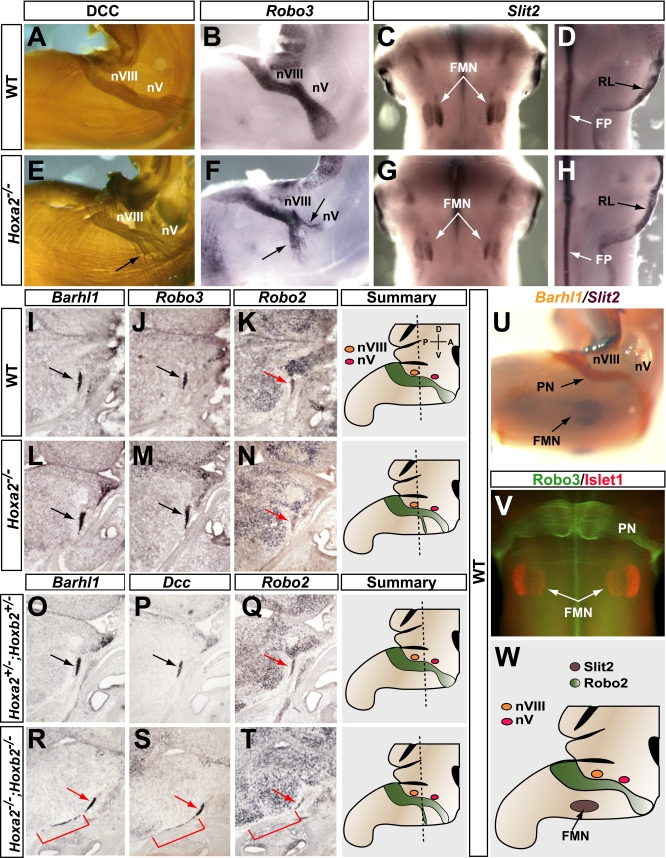
*Hoxa2*-Dependent Expression of Guidance Molecules during Pontine Neuron Migration. (A and H) Analysis of Dcc, *Robo3*, and *Slit2* expression in wild-type (WT) (A–D) and *Hoxa2^−/−^* (E–H) E14.5 whole-mount hindbrains. (A and E) Anti-DCC antibody immunohistochemistry on WT (A) and *Hoxa2^−/−^* (E) hindbrains. Dcc expression is present both in normally and ectopically (arrow in [E]) migrating PN. (B and F) Lateral views of WT (B) and a *Hoxa2^−/−^* (F) brain hybridized with a *Robo3* antisense probe. In (F), *Robo3* is expressed both in normally and ectopically (arrows) migrating PN. (C, D, G, and H) Ventral (C and G) and dorsal (D and H) views of WT (C and D) and *Hoxa2^−/−^* (G and H) brains hybridized with a *Slit2* probe. *Slit2* is expressed in the FMN (arrows in [C]), floor plate (FP), and rhombic lip (RL) (white and black arrows in [D], respectively). In *Hoxa2^−/−^* mutants, *Slit2* is down-regulated in the FMN (arrows in [G]), though not in FP and RL (white and black arrows in [H], respectively). (I–N) Adjacent cryostat sections of WT (I–K) and *Hoxa2^−/−^* (L–N) E14.5 hindbrains hybridized with *Barhl1* (I and L), *Robo3* (J and M), and *Robo2* (K and N) probes. Arrows on each panel show expression in migrating PN. In *Hoxa2^−/−^* mutants, *Barhl1* (L) and *Robo3* (M) expressions are unaffected, whereas *Robo2* (N) is down-regulated (arrow; compare K and N). (O–T) Adjacent cryostat sections of E14.5 *Hoxa2^+/−^;Hoxb2^+/−^* (O–Q) and *Hoxa2* ^−/−^;*Hoxb2^−/−^* (R–T) hindbrains hybridized with *Barhl1* (O and R), *Dcc* (P and S), and *Robo2* (Q and T) probes. In *Hoxa2* ^−/−^;*Hoxb2^−/−^* mutants, *Robo2* expression in the main PN stream is down-regulated (arrow in [T]). Note also that ectopically migrating neurons do express *Barhl1* and *Dcc* (brackets in [R] and [S]), whereas no expression of *Robo2* is detected in the ectopic stream (bracket in [T], compare with [R] and [S] adjacent sections). Summaries on the right show the distinct migratory phenotypes and planes of section shown in (I–K), (L–N), (O–Q), and (R–T). (U) Lateral view of a E14.5 WT whole-mount brain doubly hybridized with the *Barhl1* (orange) and *Slit2* (blue), showing the FMN position relative to the PN stream. (V) E15.5 WT hindbrain doubly immunostained using anti-Robo3 (green) and anti-Islet1 (red) antibodies. (W) Drawing summarizing the expression of *Robo2* (green) in migrating PN and *Slit2* (brown) in the FMN and their relative positions in respect to one another. nV, trigeminal nerve; nVIII, vestibulocochlear nerve.

To address this question, we focused on *Hoxa2* function. To achieve *Hoxa2* inactivation in PN, we mated a mouse carrying a *Hoxa2* floxed allele, *Hoxa2^flox^* [33] with the *Wnt1::Cre* transgenic mouse line that allows Cre-mediated deletion in rhombic lip progenitors [6,9,25,34]. In whole-mount brains from *Wnt1::Cre;Hoxa2^flox/flox^* fetuses, we found scattered ectopic neurons that appeared to migrate prematurely from the PN stream (Figure 3E–3H). At E15.5, the ectopic neurons had reached the ventral midline as assessed by *Tag1* expression (Figure 3G). These results supported an intrinsic requirement of *Hoxa2* expression in PN to maintain rostral migration (see also below). However, the phenotype was less pronounced than in *Hoxa2* null mutants (compare Figure 3E–3H with Figure 2D–2I). Such a difference may not be explained by an incomplete deletion of *Hoxa2* in PN precursors, as we have previously shown that the *Wnt1::Cre* driver is able to induce a complete excision of *Hoxa2* [35]. Thus, these results may rather indicate that *Hoxa2* is required both in a cell autonomous and a nonautonomous manner to regulate the response of PN to guidance cues during their rostral migration (see below; see also Figure 4).

### *Hoxa2*-Dependent Maintenance of *Robo2* and *Slit2* Expression during Pontine Neuron Migration

We next determined whether the PN migration defects detected in *Hoxa2^−/−^* mutants could be explained by a perturbed expression of ligands or receptors known to control their migration. Chemoattraction of tangentially migrating PN along the DV axis involves the Netrin-1 ligand/Dcc receptor guidance system [13–15,36] . Netrin-1 is secreted by the floor plate, whereas its receptor Dcc is expressed in PN throughout their migration (Figure 4A). In *Hoxa2^−/−^* mutant brains, we found that *Netrin-1* expression in the floor plate was unaffected (unpublished data). *Dcc* was also normally detected both in the PN stream and the ectopically migrating neurons, as shown both by in situ hybridization and antibody staining (Figure 4E and 4S; and unpublished data).

It is noteworthy that PN are not immediately attracted towards the floor plate, but undertake their long rostral migration before finally turning ventrally towards the floor plate. This suggests that, in wild-type mice, the Netrin-1/Dcc attractive signaling system may be antagonized during the rostral phase of PN migration (phase 2; Figure 1C). The chemotropic molecules of the Slit family and their Robo receptors are major repellents for developing neurons and have been shown to antagonize Netrin-1 activity on axonal growth in a dose-dependent manner [37,38]. Thus, we first investigated the spatial distribution of the *Robo1–3* receptors and *Slit1–3* ligands during PN migration. In addition to *Robo3/Rig1* (Figure 4B; [16]), *Robo1* and *Robo2* were also found to be expressed during PN migration as shown by *Robo2* in situ hybridization and by anti-Robo1 whole-mount immunostaining on E14.5 hindbrains (Figures 4K, 4Q, S1A, and S1B). The presence of Robo receptors in migrating PN was further supported by the binding on whole-mount hindbrains of a Slit2 fragment genetically fused to alkaline phosphatase (LRR-hSlit2-AP; [39]) (Figure S1C). *Slit1–3* were all expressed in the floor plate and rhombic lip, though not in PN (Figures 4C, 4D, 4U, S1D, and S1E; and unpublished data). Interestingly, from E13.0, *Slit2* and *Slit3*, but not *Slit1*, were also expressed in neurons of the FMN (Figures 4C, 4U, and S1D; and unpublished data; see below).

We next asked whether Hoxa2 may regulate *Robo* and/or *Slit* expression during PN migration. In E14.5 *Hoxa2^−/−^* fetuses, *Robo3* and *Dcc* were expressed at normal levels in migrating PN (Figure 4E, 4F, and 4M; see also Figure S3D and S3H; and unpublished data). In contrast, *Robo2* transcript levels were significantly lower in migrating PN of *Hoxa2^−/−^* single and *Hoxa2^−/−^;Hoxb2^−/−^* compound null mutants than in control mice (Figure 4N and 4T). Interestingly, down-regulation of *Robo2* expression was particularly evident in ventrally migrating ectopic cells that nonetheless maintained normal expression of *Dcc*, *Robo3*, and *Barhl1* as assessed by in situ hybridization on adjacent sections in both single *Hoxa2^−/−^* and compound *Hoxa2^−/−^;Hoxb2^−/−^* null mutants (compare Figure 4I–4K with 4L–4N, and 4O–4Q with 4R–4T). Moreover, we found a notable down-regulation of *Slit2* expression in the FMN of *Hoxa2^−/−^* mutants (*n* = 6), whereas normal *Slit2* expression levels were detected in rhombic lip and floor plate (compare Figure 4C and 4D with 4G and 4H).

Thus, the PN migratory defects observed in the absence of PG2 *Hox* function may be mediated, at least partially, through decreased Slit-Robo signaling due to defective maintenance of *Robo2-* and *Slit2*-sustained expression during PN migration along the AP axis.

### Slit-Robo–Mediated Signaling Is Required to Maintain Normal Rostral Pontine Neuron Migration

**Figure 5:**
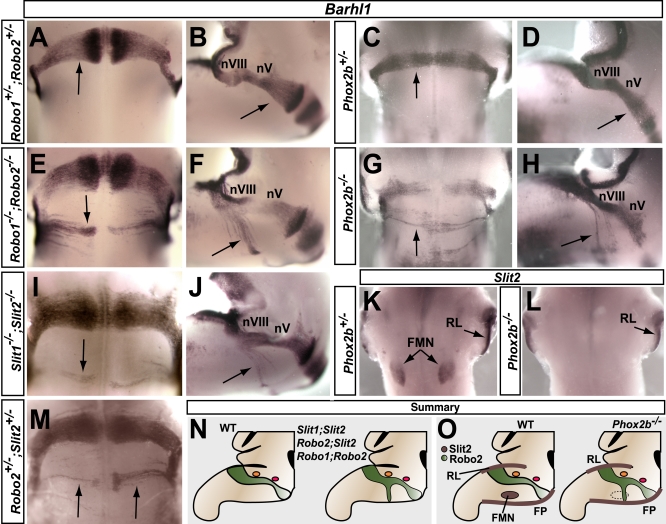
PN Migration Defects in *Robo1;Robo2*, *Slit1;Slit2*, *Robo2;Slit2*, and *Phox2b* Mutants Are Similar and Phenocopy *Hox PG2* Knockout Mice (A–J and M) Analysis of PN migration defects by in situ hybridization of whole-mount brains with a *Barhl1* probe. Lateral (B, D, F, H, and J,) and ventral (A, C, E, G, I, and M) views of *Robo1^+/−^;Robo2^+/−^* (A and B), *Phox2b^+/−^* (C and D), *Robo1^−/−^;Robo2^−/−^* (E and F), *Phox2b^−/−^* (G and H), and *Slit1^−/−^;Slit2^−/−^* (I and J), *Robo2^+/−^;Slit2^+/−^ (*M) E15.5 (A, B, E, F, I, and J) and E14.5 (C, D, G, and H) hindbrains. In *Phox2b^+/−^* and *Robo1^+/−^;Robo2^+/−^* mutants (A–D), a normal PN migratory pathway is observed (arrows). In *Robo1^−/−^;Robo2^−/−^* (E and F), *Phox2b^−/−^* (G and H), and *Slit1^−/−^;Slit2^−/−^* (I and J), and *Robo2^+/−^;Slit2^+/−^* (M) mutants, strands of ectopic *Barhl1^+^* cells migrate prematurely towards the midline and condense in small clusters at posterior locations (arrows). Such phenotypes are all similar, and phenocopy the PN migratory defects of *Hoxa2^−/−^* mutant mice (compare [E–J and M] with Figure 2D and 2E). (K and L) *Slit2* expression in *Phox2b^+/−^* (K) and *Phox2b^−/−^* (L) brains in ventral view. In (L), note the absence of *Slit2* expression at the level of the FMN due to the lack of the nucleus in *Phox2b^−/−^* mutants, whereas expression at the level of the rhombic lip (RL) is unaffected (black arrows in (K) and (L)). (N) Summary of the migration phenotypes in compound *Robo1;Robo2*, *Slit1;Slit2* deficient mice as well as in double heterozygous *Robo2^+/−^;Slit2^+/−^* . (O) Summary of the migratory defects observed in *Phox2b*-deficient mice, due to the absence of signaling from the FMN (brown on the left, dashed circle on the right). FP, floor plate; nV, trigeminal nerve; nVIII, vestibulocochlear nerve.

In *Robo3-*deficient mice, PN can still migrate along the rostral pathway (phase 2) but are unable to undergo their final ventral migration (phase 3) to reach the floor plate (Figure S2I; [16]). To address the potential involvement of *Robo1–2* and their ligands *Slit1–2* in PN migration, we next analyzed single and compound knockout mice. None of the single or compound heterozygous mutants showed significant PN migratory abnormalities (Figures 5A, 5B, and S2A–S2E). In contrast, similar migration defects of PN were observed at E15.5 in all *Robo1^−/−^;Robo2^−/−^* (*n* = 19) and *Slit1^−/−^;Slit2^−/−^* (*n* = 18) compound mutant mice, following whole-mount labeling with antisense *Barhl1* or *Robo3* riboprobes or immunostaining with anti-Robo3 (Figures 5E, 5F, 5I, 5J, S2F, and S2G; and unpublished data). Although PN normally left the rhombic lip (phase 1), during phase 2, cohorts of PN left the stream and prematurely migrated ventrally, condensing in small ectopic clusters adjacent to the midline. The leading processes of *Robo3*-expressing PN neurons were still oriented toward the floor plate and crossed it normally (unpublished data). Moreover, DiI tracing performed on E18–postnatal day (P)0 mutants showed that PN axon projections to the cerebellum were normal (unpublished data), thereby suggesting that migration, but not axon guidance, was selectively affected in these mutants. The ectopic nuclei expressed PN markers including *Barlh1*, *Pax6*, *Robo3*, and *Tag1*, and were observed at least until E17.5 (Figures 5E, 5F, 5I, 5J, S2F, and S2G; and unpublished data), as double knockouts were not viable and died a few hours after birth. The ectopic neurons were also immunoreactive for Dcc (unpublished data). However, many PN still reached a normal location in ventral r4 (Figure 5E, 5F, 5I, and 5J). Such phenotypes were fully penetrant although the size and position of the ectopic PN clusters varied between mutant embryos and brain stem side (Figures 5, S2F, and S2G). Such PN migratory defects strikingly phenocopied the abnormalities observed in *Hox* PG2 knockout animals (compare Figure 5E, 5F, 5I, and 5J with Figure 2D, 2E, 2G, and 2H). Strikingly, similar ectopic PN migrations were also observed in compound *Robo2^+/−^;Slit2^+/−^* fetuses in which only one dose of *Robo2* and *Slit2* was simultaneously deleted (Figure 5M), thus providing strong genetic evidence supporting their dosage-dependent interaction in regulating PN migration. This latter result further supports the idea that the abnormal neuronal migration observed in *Hoxa2* knockout mice may be at least partly due to the simultaneous reduction of both Robo2 and Slit2 levels (Figure 4), further underscoring the intrinsic and extrinsic requirements of *Hoxa2* in regulating rostral PN migration.

In summary, Robo1–2 and Slit1–2 molecules control in a redundant manner the horizontal, rostrally oriented migration of PN (phase 2), similar to *Hox* PG2 genes (summary in Figure 5N). However, as Slits are expressed in the floor plate throughout the AP extent of the hindbrain, the lack of expression at this location was unlikely to explain the rostrocaudal specificity of the migration defects.

### The Facial Motor Nucleus Prevents Premature Ventral Pontine Neuron Migration

The FMN, which is located in ventrolateral r6, expresses high levels of *Slit2*, and to a lesser extent *Slit3* (Figures 4C and S1D). Double immunostaining for Islet-1 and Robo3 (Figure 4V) further revealed that the rostral turn between phase 1 and 2 of PN migration coincides with the AP level of the FMN, and that PN initiate their final movement toward the floor plate (phase 3) only after they have migrated over the FMN (Figure 4U–4W). These observations, together with the results of the Slit knockouts and the reduced expression of *Slit2* in the FMN of *Hoxa2*-deficient mice, strongly suggested that the FMN may play a major role in maintaining normal horizontal migration of PN through expression of Slits.

To further support the potential involvement of the FMN in PN horizontal migration, we analyzed a mouse mutant devoid of cranial branchiomotor nuclei. The paired homeodomain-containing *Phox2b* gene is required for the generation of all branchiomotor neurons [40]. *Phox2b* inactivation resulted in the lack of all cranial branchiomotor nuclei, including the absence of the FMN as confirmed by the lack of *Islet1* and *Slit2* staining on whole-mount E14.5 *Phox2b^−/−^* specimen (Figure 5L; [40]; and unpublished data). In contrast, *Slit2* was normally expressed in the rhombic lip and floor plate of *Phox2b^−/−^* mutants (Figure 5L; and unpublished data). In E14.5 *Phox2b^−/−^* embryos, cohorts of PN prematurely migrated ventrally and generated small supernumerary nuclei in ectopic posterior locations (Figures 5G, 5H, and S2H; *n* = 4) as in *Hox* PG2, *Robo1^−/−^;Robo2^−/−^*, *Slit1^−/−^;Slit2^−/−^*, and *Robo2^+/−^;Slit2^+/−^* mutant mice (compare Figures 2 and 5).

Overall, these results strongly indicated that the FMN is an important extrinsic source of Slit signaling for rostrally migrating PN to prevent their premature ventral migration (summary model in Figure 5O).

### *Robo2* Is a Direct Target of Hoxa2

**Figure 6:**
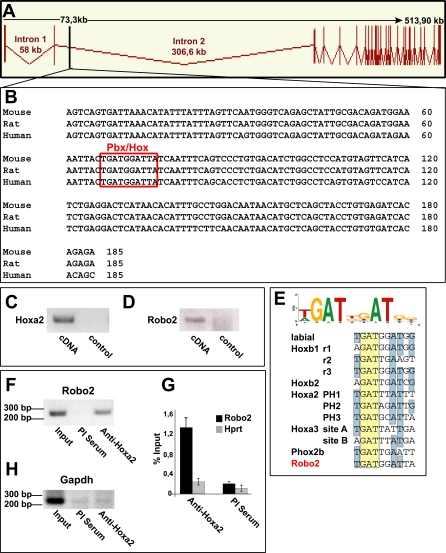
*Robo2* Is a Direct Target of Hoxa2 (A) Schematic representation of the *Robo2* locus. Vertical and horizontal lines represent exons and introns, respectively. (B) A highly conserved 185-bp fragment, in the second intron 73.3 kb downstream of the *Robo2* transcription start site (Chromosome 16: 74221061–74221245 UCSC Genome Browser), contains a putative Pbx/Hox binding site (red box). (C and D) Transcript detection from *Hoxa2* (C) and *Robo2* (D) coding regions by RT-PCR of total RNA retrotranscribed (cDNA) from P19 cells. The control (C) is carried out on nonretrotranscribed total RNA. (E) On top: consensus sequence used to screen the *Robo2* locus for Pbx/Hox binding sites. Below are aligned the identified Pbx/Hox sites (red) in the *Robo2* locus with known Pbx/Hox binding sites present in other *Hox* target genes. Complete conservation of nucleotides among the different sites is shown in yellow. In blue are shown nucleotide positions with high degree of conservation. (F–H) ChIP assay performed with a specific rabbit anti-Hoxa2 polyclonal antibody (Anti-Hoxa2) on total chromatin (Input) from P19 cells, as detailed in experimental procedures. In the experiment shown in (F), a specific PCR product spanning the identified Pbx/Hox site in *Robo2* is amplified from immunoprecipitated chromatin with the anti-Hoxa2 antibody, though not with the rabbit preimmunized (PI) serum. (H) Control PCR showing the lack of specific amplification products from the same immunoprecipitated material as in (F) when using specific primers within the *Gapdh* locus. (G) Quantitative PCR (qPCR) from ChIP assays independent from (F and H). The values from the LightCycler are expressed as a percentage of the total input. A highly significant enrichment is observed for the fragment spanning the *Robo2* Pbx/Hox site (black bar) from the anti-Hoxa2 immunoprecipitated chromatin (left), as compared to an *Hprt* control genomic fragment (grey bar). To the right is shown the percentage of the input recovered using the control rabbit preimmunized (PI) serum. The data are presented as the mean ± standard error (S.E.) and are from two independent amplifications, each performed in duplicate.

In *Drosophila* embryo, *Robo2* expression in the mesoderm is likely to be controlled by Hox cofactor genes such as *homothorax* [41]. In addition, a putative *Hox* binding site has been described in the *Drosophila Robo2* locus, although evidence of direct regulation is lacking [41]. To investigate whether Hox PG2 factors may directly regulate some aspects of the Slit-Robo signaling system in the mouse, we tested the ability of Hoxa2 to bind the *Robo2* regulatory genomic region in vivo. As Hox proteins preferentially bind their target genes through heterodimerization with Pbx cofactors (e.g., [42,43]), we screened in silico about 500 kb of genomic sequence containing the entire *Robo2* locus for the presence of potential NGATNNATNN Pbx/Hox consensus binding sites (Figure 6E; [44–47]). We only considered potential sites that were embedded within 150–500 base pair–long DNA stretches, displaying more than 90% nucleotide conservation at the nucleotide level in mammals and other vertebrates (Figure 6B; and unpublished data). By applying such constraints, we selected four putative Pbx/Hox binding sites located within 10 kb upstream of the *Robo2* transcription start site, as well as within the first and second introns (Figure 6A and 6B; and unpublished data).

To test the potential of Hoxa2 to bind *Robo2* in vivo, we performed chromatin immunoprecipitation (ChIP) analysis [48] on the selected sites from P19 teratocarcinoma cells, a suitable cell culture system to study Hox-regulated targets (e.g., [43]). Indeed, P19 cells expressed significant levels of *Robo2* and *Hoxa2*, as detected by reverse transcriptase PCR (RT-PCR) (Figure 6C and 6D). To perform ChIP on putative Hoxa2 binding sites, we generated a specific polyclonal antibody raised against a unique peptide of the Hoxa2 protein (see Materials and Methods). Nonquantitative PCR amplification of DNA fragments containing the four putative binding sites was carried out on anti-Hoxa2 immunoprecipitated chromatin. As shown in Figure 6F, the immunoprecipitated chromatin showed a substantial enrichment selective for the sequence, including the site located in the second intron. No enrichment was detected for the remaining sites, as well as for the control *Gapdh* gene (Figure 6H; and unpublished data). To further support these data, we carried out real-time quantitative PCR (qPCR) assays on immunoprecipitated chromatin with the anti-Hoxa2 antibody. A strong enrichment of the fragment containing the putative Pbx/Hox binding site in the second intron was confirmed, as compared to controls (Figure 6G).

Altogether, these results demonstrated that Hoxa2 can directly bind *Robo2* genomic sequences in vivo and, together with the results in knockout animals, strongly suggested that Hoxa2 may directly regulate sustained *Robo2* expression during PN migration.

## Discussion

### *Hox* Gene–Dependent Control of Pontine Neuron Migration along the Anteroposterior Axis

The LRN, ECN, RTN, and PGN constitute the major sources of cerebellar mossy fibers [3]. These nuclei originate from the same stripe of rhombic lip neuroepithelium in the posterior hindbrain, and their generation periods partially overlap [4,10,49,50]. Neurons of these nuclei also express a similar set of transcription factors before leaving the rhombic lip, including *Pax6* and *Math1* [51], and during their migration and settling, such as *Pax6* and *Barhl1* [21,32]. Expression of such transcription factors may provide cells with information about their specification as mossy fiber precerebellar neurons and/or to acquire a general migratory behavior upon exiting the rhombic lip. Accordingly, precerebellar neurons migrate abnormally in *Pax6* and *Barhl1* knockout mice [21,32]. Yet, neurons contributing to distinct nuclei migrate following specific pathways and settle at stereotypic AP and DV positional coordinates in the brain stem. Thus, other sets of transcription factors must regulate the responsiveness of migrating precerebellar neurons to environmental guidance cues and drive their distinct migratory routes.

*Hox* genes are prime candidates to regulate the directionality of cell migration along the AP axis, although so far little evidence has been available in the mammalian central nervous system. We found that PN contributing to RTN and PGN expressed *Hox* PG2–5 genes throughout their AP migration and settling, thus expressing a code characteristic of their axial origin posterior to r5. Segmental specification and the *Hox* expression program of precerebellar neurons may select which migratory direction to take upon leaving the rhombic lip. In Caenorhabditis elegans, the rostral or caudal migratory choices of the QR or QL neuroblasts are regulated by the expression of the *lin-39* and *mab-5 Hox* genes, respectively [52]. In the chick embryo, overexpression of Hoxa2 in the r1 rhombic lip, normally devoid of *Hox* expression, induced neuronal derivatives to migrate ventrally instead of rostrally, thereby adopting a migratory route reminiscent of more-posterior rhombic lip derivatives [53]. Here, we show that mouse PG2 *Hox* genes are involved in the maintenance of the rostral migration of PN by preventing premature ventral migration and settling of PN at posterior locations. Similarly, specific *Hox* programs might control the directionality of migration of other precerebellar nuclei along the AP axis. Analysis of the specific *Hox* expression codes of all precerebellar nuclei will be required to support such an hypothesis.

An interesting finding is the variability in the penetrance and/or severity of the migratory phenotypes in *Hoxa2* or *Hoxb2* mutants. In *Hoxa2* mutants, the fraction of PN displaying migration errors varied both in spatial distribution and number among individuals, whereas the bulk of PN followed a normal rostral migration pathway (Figure 2). We also often observed asymmetric phenotypes between the two sides of the same brain stem. Furthermore, in *Hoxb2* mutants, only one third of the specimen displayed an abnormal phenotype. The lack of specific molecular markers did not allow us to distinguish between reticulotegmental or pontine gray neuron identities within the ectopic PN subpopulation. Nonetheless, overall, our results strongly suggested that the ectopic neurons belonged to a random subpopulation due to insufficient redundant functional gene effects. On the one hand, such differences in phenotypes indicate locally random variations of threshold levels of guidance cues and/or of responses of PN in mutants. On the other hand, the limited extent of the abnormal migratory phenotype indicates that such a molecular guidance system is quite robust and buffered against a certain degree of variation, such as loss of function of one or two *Hox* genes. In fact, even in double PG2 *Hox* mutants, many PN still migrated normally (Figure 2). Thus, the loss of PG2 *Hox* function may be stochastically compensated by other members of the *Hox* family during PN migration through rhombomeric domains.

In a strict interpretation of the “posterior prevalence” model of Hox function [54], only the most “posterior” (i.e., 5′ located in the cluster) PG *Hox* gene expressed, i.e., PG5, would be expected to select the migratory behavior of PN. The results of PG2 *Hox* gene inactivations indicated that such a strict model is unlikely to be operating in migrating PN. Rather, a partially quantitative aspect may be added in which local guidance responses of PN may also rely on overall Hox protein distribution and/or levels. Alternatively, a “dynamic” posterior prevalence model might be at work in which the preponderant role of PG5 to PG2 may be sequentially switched while PN are progressing rostrally across inter-rhombomeric domains. The program of *Hox* gene expression in migrating neurons may continuously integrate extrinsic segment-specific cues with intrinsic regulation of relevant target gene expression, instructing guidance information to PN about the progression of their positional coordinates along the AP axis. Discriminating between such possibilities will need to await the analysis of PN migration in single and compound knockouts for PG3–5 *Hox* genes.

### Autonomous and Nonautonomous Requirements of PG2 *Hox* Genes to Regulate Responsiveness of Migrating Pontine Neurons to Guidance Cues

We show that the integrity of the *Hox* expression program of PN during migration is required to regulate their responsiveness to guidance cues and that *Hox* PG2 genes are important components of such a molecular guidance system. Specifically, the analysis of *Hoxa2* knockout animals supported that *Hoxa2* is required both intrinsically in PN and extrinsically to define a local environment permissive to their rostral migration. This assumption is based on the following observations: (1) *Hoxa2* is expressed throughout migration and settling of PN (Figure 1); (2) in *Hoxa2* mutants, early steps in migration appear unaffected; this suggests that migration itself, rather than an early event such as the generation of precursors fated to form the pontine nucleus, is being affected; (3) the inactivation of *Hoxa2* in *Wnt1^+^* precerebellar precursors resulted in migration errors of PN (Figure 3), although at a lower penetrance and severity than in the null mutants, suggesting that *Hoxa2* may be additionally required to pattern the environment through which PN migrate; and (4) in *Hoxa2* mutants, the expression of *Robo2* in migrating PN and *Slit2* in FMN are down-regulated, and compound *Robo2^+/−^;Slit2^+/−^* mutants showed that normal expression levels of such molecules are required for PN guidance (Figures 4 and 5).

Thus, the PN migratory phenotype observed in *Hoxa2^−/−^* mutants is likely to result from an impairment of *Hoxa2* function both in migrating neurons and in the local environment through which PN migrate.

### Maintenance of Pontine Neuron Migration along the Anteroposterior Pathway Requires Slit-Robo Signaling

Little is known about how signaling mediated by distinct guidance molecules distributed along the DV and AP axes is integrated in PN during their tangential migration. Our data suggest that specific molecules control PN migration behavior at precisely defined choice points and that Slit/Robo signaling plays a key role in this process.

Although numerous studies have involved Slit-Robo ligand–receptor interaction in axon guidance and branching [2,39,55–61], much less is known about their involvement in neuronal migration, in particular in vivo (e.g., [59,62–64]). For instance, it was previously shown in mice that the Robo3 receptor is required for the last phase of ventral PN migration (Figure S2; [16]). In mice lacking *Robo3*, PN still leave the rhombic lip and migrate rostrally, but are unable to turn towards the ventral midline, despite normal expression of *Netrin-1* and *Dcc* (Figure S2; [16]; and unpublished data). Robo3 was proposed to function as a negative regulator of Slit responsiveness, somehow repressing Slit repulsive activity from the floor plate and thus interfering with Robo1/Robo2 receptor activation [65]. *Robo3* is coexpressed with *Robo1* and *Robo2* during PN migration until they reach the floor plate (Figures 4B and S1), when *Robo3* expression is down-regulated. Thus, one possibility is that in *Robo3*-deficient mice, Robo1/Robo2 repulsive activity would be activated too early, unmasking Slit repulsive activity from the floor plate and forcing PN to remain in the dorsal hindbrain. According to this model, midline-derived Slit would be the main repulsive source for PN neurons (but see below).

In addition to the floor plate source, Slits are expressed at the rhombic lip. Although migrants from the rhombic lip have been shown to be repelled by exogenous sources of Slit2 in coculture [66], our present data indicated that Slit-mediated repulsion may not control the phase 1 of PN migration from the rhombic lip, as PN still leave the rhombic lip in compound *Slit1;Slit2-*, *Robo1;Robo2-*, and *Robo2;Slit2*-deficient mice. However, *Slit3* might still compensate for the loss of *Slit1/Slit2* expression at the rhombic lip. Instead, our analysis revealed a major role for Slit1/Slit2 and Robo1/Robo2 during the phase 2 of PN migration along the AP axis and identified the FMN as another important source of Slits for migrating PN, in addition to the floor plate (see also below). Impaired Slit/Robo signaling resulted in strands of PN migrating out from the stream along the DV pathway in ectopic posterior positions above the FMN (Figure 5). Thus, Slit2-mediated signaling from the FMN and Slit1/Slit2 from the floor plate are among the main driving forces that prevent PN from reaching the ventral midline upon leaving the rhombic lip, forcing them to migrate rostrally towards r3. The fact that in compound *Slit1;Slit2* and *Robo1;Robo2* mutants, the leading process of PN still cross the floor plate and then project into the cerebellum (unpublished data) strongly suggests that in this system, Slit/Robo signaling primarily controls cell migration and not axon guidance.

The DV PN migration requires floor plate-derived Netrin-1 and its receptor Dcc [14,15]. However, even in the absence of *Netrin-1* or *Dcc*, PN manage to undertake phase 1 and phase 2 of migration, leaving the rhombic lip and navigating rostrally before aggregating in an ectopic dorsal position [15]. Thus, attraction by Netrin-1 towards the midline appears to be essential only during phase 3 of migration, once PN turn ventrally towards the floor plate. As a corollary, PN must be partially insensitive to the Netrin-1/Dcc attraction before reaching ventral r4, despite their continued expression of Dcc (Figure 4A) and the presence of Netrin-1 all along the floor plate [15].

Our results show that in the absence of Slit1–2- or Robo1–2-mediated signaling, many PN migrate prematurely towards the midline. This suggests that Slit repulsive activity may counterbalance and prevail over Netrin-1/Dcc–mediated attraction. Slit-Robo signaling may negatively regulate the responsiveness of PN to Netrin-1/Dcc through several possible mechanism(s). For instance, the activation of Robo1/Robo2 receptors in migrating PN by secreted Slit ligands might lead to dimerization of the intracellular domains of Robo and Dcc, resulting in a partial silencing of Netrin-1 attraction on PN, as described in *Xenopus* spinal axons [38]. Thus, in such a scenario, the activity of Slit-activated Robo receptors would be required to inhibit Dcc activity in target neurons. However, Slit/Robo and Netrin-1/Dcc guidance systems could also be acting independently, and PN migratory behavior could result from a balanced integration of attractive and repulsive responses within target neurons. Such possibilities are not mutually exclusive and remain speculative at this point.

**Figure 7:**
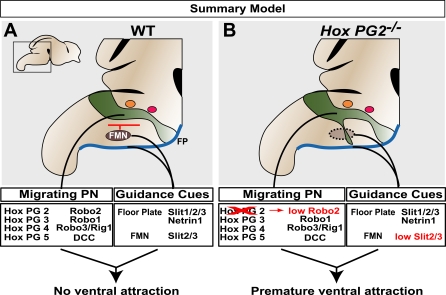
Summary Model for the Control of Rostral Pontine Neuron Migration by *Hox PG2* Genes, Guidance Molecules, and the Facial Motor Nucleus. (A) Migrating PN express PG2–5 *Hox* genes as well as Robo1–3 and Dcc receptors. The ligands Slit1–3 and Netrin-1 are expressed throughout the hindbrain floor plate (FP). Moreover, Slit2 and Slit3 are expressed in the FMN (brown). Based on this work, during the PN rostral phase of migration, the Netrin-1/Dcc–mediated ventral attraction may be antagonized through Slit-Robo signaling, thereby preventing a premature ventral migration of PN. In addition, the FMN is an important signaling source required to maintain the caudorostral PN migratory pathway (red bar), partly through Slit2–3 expression. (B) In *Hox* PG2 mutant mice, *Netrin-1* and *Slit1–3* expression at the FP level as well as *Dcc* expression within migrating PN are not affected. However, *Hoxa2* is required to maintain sustained *Robo2* expression within migrating PN, and normal *Slit2–3* expression within the FMN (dashed circle). Insufficient Slit-Robo signaling in turn led to the inability of some PN to maintain their normal rostral pathway, resulting in ectopic neurons prematurely migrating ventrally at posterior locations. Such behavior of migrating PN is phenocopied in compound *Robo1;Robo2-*, *Slit1;Slit2-*, *Robo2;Slit2*-deficient mice as well as in FMN-deficient *Phox2b* mutant mice.

It is noteworthy that single *Robo1*, *Robo2*, *Slit1*, or *Slit2* homozygous mutant mice did not show significant PN migration defects (Figure S2). This argues for redundant functional roles among Robo receptors or Slit ligands. However, the PN migratory defects in compound *Robo1;Robo2* mutants phenocopied those of compound *Slit1;Slit2* mutant mice, showing that both Robos and Slits are required for PN migration. This strongly favors a direct Slit/Robo interaction in migrating PN. Strong support for this idea also came from the analysis of compound Robo2^+/−^;Slit2^+/−^ heterozygotes in which the deletion of only one dose of Robo2 or Slit2 was sufficient to induce migration defects similar to those observed in double Robo1–2- or Slit1–2-deficient mice, demonstrating Slit/Robo dose-dependent interactions. Moreover, from the results of *Slit1;Slit2* compound mutants and the analysis of *Phox2b* knockout mice lacking the FMN (Figure 5), it can be inferred that the Slit2 source diffusing from the FMN is necessary to maintain the normal rostral migration of PN neurons (models in Figures 5O and 7A). At r3 level, PN neurons may turn ventrally because Slits diffusing from the FMN in ventral r6 may become limiting and fall below a threshold level. Notably, it is also at this axial level that Robo3 function becomes preponderant and interferes with Slit repulsive activity.

Finally, our results indicate that neither Robos nor Slits are essential for the anterior progression towards r3 of PN, but rather that they prevent migrating neurons from entering the wrong territories. Additional signals must be involved in attracting PN anteriorly and/or repelling them from the posterior brain stem. Attractive signals from trigeminal branchiomotor neurons (MN) can be ruled out because PN still migrate in *Phox2b* knockout animals, which lack all branchiomotor nuclei, including trigeminal MN (Figure 5G and 5H). Another possibility is that PN might use the adjacent trigeminal nerve tract as a migration substrate to orientate themselves. Neurons from the posterior migratory stream have been shown to adopt such an axonophilic migration [67]. Chemoattraction of PN could also be provided by the meninges that overlay the migrating stream. Signaling from the meninges has been implicated in the tangential migration of cortical hem-derived Cajal-Retzius cells in the cerebral cortex. The meninges secrete the chemokine CXCL12 and enhance the migration of CXCR4 expressing Cajal-Retzius cells [68]. Interestingly, migrating PN express CXCR4, and their migration is disrupted in CXCR4-deficient animals [69]. Moreover, CXCR4/CXCL12 signaling can be modulated by Slit upon Robo binding to CXCR4 [70]. Lastly, the meninges over the migrating PN have been shown to be a localized source of retinoic acid, and treatment of the fetus with exogenous sources of retinoic acid has been shown to result in migratory abnormalities of precerebellar neurons [71].

### The Role of the Facial Motor Nucleus in Pontine Neuron Migration

Our data indicate a novel role for the FMN in maintaining the rostral migration of PN. In mammals, facial motor neurons migrate tangentially from the ventricular region of r4 across r5 to colonize r6, where they undergo a radial migration to finally condense into the FMN, next to the pial side in ventral r6 [72–74]. Such a stereotyped migration takes place between E10.0 and E14.0, at which stage most of the facial motor neurons have reached their final destination in ventral r6 [73]. In addition to PN, the r6–r8 rhombic lip neuroepithelium generates the ECN and LRN neurons that undertake a ventrally oriented extramural pathway to finally settle in the caudal brain stem. ECN/LRN start migrating earlier than PN, approximately between E13.0 and E15.0, whereas PN migration occurs between approximately E13.5 and E17.5 [10,50]. PN and ECN/LRN neurons express similar molecular markers, including Robo2 and Dcc receptors, yet they display distinct migratory pathways and final locations [9,10,36,49,51]. There is a temporal correlation between the end of the posterior FMN migration and the beginning of the PN rostral migration (phase 2). Thus, one possible mechanism to maintain the distinct migratory behavior of these populations could depend on the timing of Slit repulsive signaling from the FMN. Specifically, by the time most of the facial motor neurons have reached their final location in ventral r6, i.e., around E14.0, the amount of Slit secreted from the FMN could become quantitatively sufficient to antagonize the Netrin-1/Dcc–mediated attraction. This may prevent further ventral progression of neurons along the ECN/LRN pathway and allow later-born neurons, at least those closer to the source, to maintain a rostral PN pathway. An additional role might be played by the Slit-expressing facial motor axons exiting the hindbrain at the level of r4. Indeed, the PN stream has to navigate in a “corridor” delimited dorsally by the VIIIth and ventrally by the VIIth nerve roots (Figure 1; [25]). Thus, decrease of Slit-mediated repulsion at the level of the VIIth nerve root might also contribute to the PN navigation errors observed in the various mutants.

Notably, the migratory behavior of the FMN varies in different vertebrate species due to specific signaling cues in r5 and r6 (e.g., [74]). Sharks, lizards, or salamanders have similar organization and location of FMN to mammals, whereas in zebrafish, facial motor neurons migrate into r6 and r7 [75]. Chick embryos are peculiar in that facial motor neurons migrate dorsally within r4, similarly to trigeminal branchiomotor neurons in r2. Interestingly, rhombomere contributions and migratory pathways of pontine nuclei also vary among vertebrates (e.g., [8]). Thus, one possibility is that the distinct migratory behavior of the FMN in different vertebrates may in turn contribute to explain distinct species-specific migratory routes of PN.

Finally, based on our results, it is likely that structure(s) other than the FMN are involved in signaling to PN. Indeed, in *Phox2b* mutants, the migration errors induced by the lack of FMN are only partial, whereas the bulk of PN follow their normal pathway of migration (Figure 5G and 5H), thus indicating the influence of additional structure(s). Also, in compound *Slit1;Slit2* mutant mice, the expression of Slit3 in FMN is not sufficient to rescue the absence of Slit2, also resulting in a partially penetrant migration phenotype similar to the *Phox2b* mutant phenotype (Figure 5I and 5J). Hence, whereas our analysis does not allow us to determine the exact location of the Slit1 source, a combinatorial activity of Slit molecules from the FMN and other sources such as the floor plate appears necessary to maintain the rostral migration of PN.

### Hoxa2 Controls Pontine Neuron Migration through Direct Regulation of *Robo2*

To date, only a handful of direct targets of *Hox* genes have been identified in vertebrates [42,43]. In particular, despite the increasing evidence for *Hox* gene expression during late phases of mammalian nervous system development (e.g., [20]), the nature of Hox direct targets remains largely elusive. Thus, it is unclear how *Hox* genes may contribute to the molecular regulation of complex aspects of neural circuit assembly, such as neuronal migration and/or topographic axon pathfinding.

In the case of *Hox* PG2 genes, we show that their main role is to maintain the migration of the PN along a defined AP pathway. Interestingly, this is achieved not by direct negative regulation of Netrin-1/Dcc, but via the local modulation of Slit-Robo signaling levels while PN migrate through distinct rhombomeric territories. Another important finding is that the regulation of genes encoding for transcription factors involved in general aspects of PN differentiation or migration such as *Barhl1* or *Pax6* was not affected by the lack of *Hox* PG2 genes, indicating that regulation of PN directional migration by *Hox* genes act in parallel with or downstream from such transcription factor activities (Figure 2; and unpublished data).

As for Hoxa2, our ChIP assays in P19 cells and in situ hybridization data on mutant fetuses strongly support direct regulation of *Robo2* expression levels in migrating PN, whereas regulation of *Slit2* expression in the FMN appears to be indirect. In fact, at the time when *Slit2* down-regulation is observed, *Hoxa2* is not expressed in facial branchiomotor neurons (unpublished data). Generation, migration, and initial differentiation of facial branchiomotor neurons appear to take place normally in *Hoxa2* mutants (unpublished data), although an early patterning defect of r4- and/or r5-derived motor neurons cannot be formally ruled out. Down-regulation of *Slit2* in the absence of Hoxa2 might be more likely due to abnormalities in r4 neural crest-derived glia resulting in a late impairment of FMN maintenance and connectivity. Indeed, lack of *Hoxb1* in r4 neural crest-derived glia resulted in late FMN defects similar to in *Hoxa2* knockout animals [76]. In addition, in *Hoxa2^−/−^* mutants, the identity of second branchial arch-derived muscles targeted by the facial nerve is altered, potentially resulting in a late degeneration of the FMN [30].

### Conclusions

In conclusion, we provide for the first time, to the best of our knowledge, evidence for the implication of *Hox* PG2 genes in tangential migration of PN and identify the guidance receptor *Robo2* as a direct target gene of *Hoxa2*. We further show that Slit-Robo signaling involving sources of Slit2 from the facial branchiomotor nucleus and Robo2 expression in PN are key components of the molecular guidance system maintaining PN rostral migration. Our data provide a conceptual framework to understand how the PN response to multiple guidance cues along the AP and DV axes is regulated at the transcriptional level and in turn translated into coherent neuronal migratory behavior (see model in Figure 7).

## Materials and Methods

### Mouse line generation and genotyping.

*Slit-* and *Robo*-deficient mice were previously described and genotyped by PCR [39,56,60]. *Hoxa2* null and *Hoxa2^flox^* lines were previously described and genotyped by PCR [30,33]. The day of the vaginal plug was counted as E0.5. All animal studies were done in accordance with the guidelines issued by the French Ministry of Agriculture.

### Rescue of *Phox2b^−/−^* embryo lethality.

*Phox2b^−/−^* homozygous mutant embryos die at E10.5–E13.5 [40]. However, treatment with noradrenergic agonists allows the rescue of embryo lethality through later stages. To obtain *Phox2b^−/−^* fetuses, drinking water of pregnant *Phox2b^+/−^* females was supplemented with 1 mg/ml of l-phenylephrine, 1 mg/ml of isoproterenol, and 2 mg/ml of ascorbic acid, from E8.5 onwards. Mutant fetuses were genotyped as described in [40].

### Binding with LRR2-hSlit2-AP.

To generate the human Leucine Rich Repeat (LRR) Slit2-alkaline phosphatase fusion protein (LRR2-hSlit2-AP), the second LRR of Slit2 (amino acids 341–505) was amplified by PCR and cloned between the XhoI and XbaI sites of AP-Tag5 vector (Genhunter). Binding was performed as described in [39].

### Immunohistochemistry.

After dissection, brains were fixed by immersion in 4% paraformaldehyde in 0.12 M phosphate buffer (pH 7.4) (PFA). Brains were blocked in 0.2% gelatin in PBS containing 0.25% Triton-X100 and incubated overnight at room temperature with goat anti-rat Robo1 (R&D Systems), goat anti-human DCC (Santa Cruz Biotechnology), goat anti-human ROBO3 (R&D Systems), and rabbit anti-Islet 1 (Abcam), followed by species-specific biotin-coupled secondary antibodies (Jackson Laboratories) or fluorochrome-coupled secondary antibodies donkey anti-goat CY3 (Jackson Laboratories) and donkey anti-goat A488 (Invitrogen). Detection was performed using Vectastain *Elite* ABC Kit following manufacturer instructions.

### In situ hybridization.

In situ hybridization was performed as described in [16]. Briefly, brains were dissected, fixed in 4% paraformaldehyde (PFA) overnight, cryoprotected in 20% sucrose, and then embedded in Shandon Cryomatrix (Thermo Electron Corperation) before freezing at −80 °C. The 20-μm cryostat sections were cut in a coronal plane. For whole-mount staining, brains were fixed in 4% PFA overnight, dehydrated, and then stored at −20 °C in 100% methanol. For double in situ hybridization analyses, antisense riboprobes labeled with fluorescein-11-d-UTP were additionally used.

Whole-mount hindbrains were processed as for single in situ hybridization [16], but the two antisense riboprobes labeled with digoxigenin-11-d-UTP (*Barhl1*) or fluorescein-11-d-UTP (*Slit2*) were mixed (200 ng/ml) in the hybridization buffer. The Dig-UTP probes recognized by an anti-DIG antibody conjugated to alkaline phosphatase, was detected first by NBT-BCIP reaction. Next, hindbrains were rinsed in a solution of glycine (0.1 M [pH 2.2]) during 15 min and extensively washed with MABT (pH 7.5) (NaOH 200 mM, maleate 100 mM, NaCl 150 mM, Tween 20 0.1%). They were blocked in a solution of Tris (pH 7.6) 100 mM, NaCl 150 mM, Tween 0.1%, complemented with 20% normal goat serum (NGS), for 1 h at room temperature. Hindbrains were incubated overnight at 4 °C with anti-fluorescein antibody conjugated to alkaline phosphatase (1/5,000; Roche Diagnostics). After several washes, the alkaline phosphatase activity was detected using INT (2-[4-iodophenyl]-3-[4-nitrophenyl]-5-phenyltetrazolium chloride) (2.48 mg/ml) and BCIP (5-bromo-4-chloro-3-indolyl phosphate, toluidine salt in DMSO) (2.48 mg/ml; Roche Diagnostics) diluted in a solution of Tris (pH 9.5) 100 mM, NaCl 100 mM, MgCl_2_ 50 mM, Tween 0.1%, levamisole 2 mM. Whole-mount hindbrains were fixed overnight in 4% PFA and stored at 4 °C in PBS/70% glycerol. The following probes were used: *Hoxa2* [30]; *Hoxb2* [77]; *Hoxa3* [78]; *Hoxb3* [79]; *Hoxb4* [80]; *Hoxd4* [81]; *Hoxb5* [82]; *Barhl1* [21], *Pax6* [83], and *Tag1* [31].

### Alkaline phosphatase staining.

For alkaline phosphatase staining, brain sections were incubated in PBS at 65 °C for 90 min to inactivate the endogenous alkaline phosphatase. After the inactivation, sections were washed in a solution of NTMT (NaCl 5 M, Tris HCL 1 M [pH 9.5], MgCl_2_ 1M, and Tween 20 50%). Finally, the sections were overlaid in a solution consisting of NTMT with NBT and BCIP. The sections were rinsed first in PBS and next in EtOH 100%, and then mounted onto slides.

### LacZ staining.

Whole embryos were processed for β-galactosidase histochemistry by two washes in 0.1 M phosphate buffer (pH 7.4), fixed in 4% PFA at 4 °C for 1 h. After two washes in 0.1 M phosphate buffer, brains were put into the staining solution (2 mM MgCl_2_, 5 mM K_3_Fe(CN)_6_, 5 mM K_4_Fe(CN)_6_, Tween 20 0.1%, PFA 0.2%, and 1 mg/ml X-gal) at 37 °C for 2 h. Stained brains were then dehydrated in methanol 100%, and stored at −20 °C for further analysis by in situ hybridization.

### RT-PCR.

Total RNA from P19 cells was collected using Trizol reagent following manufacturer protocol (GibcoBRL). cDNA synthesis was performed using SuperScriptII Reverse Transcriptase (GibcoBRL) following the manufacturer's instructions. Primers: *Hoxa2*: PCR1: 5′-TCGACGCTTTCACACTCGACACTGAT-3′ (forward) and 5′-CCGGTTCTGAAACCACACTT-3′ (reverse). PCR2 (nested): 5′-AGTCACCCTCGCCACGGCGCT-3′ (forward) and 5′-TCTGCAAAGGTACTTGTTGA-3′ (reverse). *Robo2*: 5′-ATATCTGATACTGGCACTTATAC-3′ (forward) and 5′-CTGAAAGCCTCAATGATATACGC-3′ (reverse).

### Anti-Hoxa2 antibody generation.

An anti-Hoxa2 rabbit polyclonal antibody was raised by immunization against the following peptide: KFKNLEDSDKVEEDEEEKSLC, encompassing amino acids 209–228 of the Hoxa2 murine protein. Such a unique peptide was chosen to avoid potential cross-reactivity of the antibody with other Hox proteins. Before injection, the peptide was coupled to activated ovalbumin according to the manufacturer's protocol (Pierce). Antibody specificity was tested on Western blot from extracts of COS cells transfected with Hoxa2 or other Hox proteins (unpublished data).

### Chromatin immunoprecipitation assays.

ChIP assays were performed using the Upstate EZ ChIP Chromatin immunoprecipitation Kit, following the manufacturer's protocol. P19 cells were cultured to confluence, fixed in 1% formaldehyde, and sonicated 24 times for 10 s in 50-s intervals using a Fischer Bioblock Scientific Sonicator (Vibracell). Sonicated DNA was immunoprecipitated using the generated polyclonal anti-Hoxa2 antibody and control antibodies. The immunoprecipitated *Robo2*, *Gapdh*, and *Hprt* sequences were first amplified and detected by nonquantitative PCR (31 cycles) followed by visualization on agarose gel. In addition, real-time quantitative PCR (qPCR) was carried out using the Quantitech SYBR Green PCR Kit (QIAGEN) with a Roche LightCycler. The ChIP primers for nonquantitative PCR are as follows: *Robo2*: 5′-CTATGGGTTTTGCTTTATCTGTCCC-3′ (forward) and 5′-GGTAGCTGAGCATGTTATTGTCC-3′ (reverse); *Gapdh*: 5′-TCTGCGCCCTTGAGCTAGGACTGG-3′ (forward) and 5′-TTCGCACCAGCATCCCTAGACC-3′ (reverse). For real-time qPCR, the following oligonucleotide primers were used: *Robo2*: 5′-TGATAAGTTGACCAGTCAGTG-3′ (forward) and 5′-TGTGTTATGAGTCCTCAGATG-3′ (reverse); *Hprt*: 5'-TTATCTGGGAATCCTCTGGG-3′ (forward) and 5′-AAAGGCAGTTCCGGAACTCT-3′ (reverse).

## Supporting Information

Figure S1 Expression Patterns of *Robo1*, *Robo2*, and *Slit3*
(A) Lateral view of a E14.5 wild-type (WT) whole-mount brain, immunostained with anti-Robo1 antibody. The black arrow shows the presence of Robo1 in migrating PN.
(B) Ventral view of a E15.5 WT whole-mount brain hybridized with a *Robo2* probe. *Robo2* is expressed within PN throughout their migration and maintained within the nascent PGN (red arrows).
(C) LRR2-hSlit2-AP binding on a E14.5 WT whole-mount brain (lateral view). The arrow indicates the presence of Slit2 receptors in migrating PN.
(D) Expression of *Slit3* in the FMN (white arrow) of a E14.5 whole-mount mouse brain.
(E) Dorsal view of one side of the same brain shown in (D) (midline to the left, dorsal side to the right) showing *Slit3* expression in the rhombic lip (black arrow), as well as in the floor plate (white arrow).
FP: floor plate; nV: trigeminal nerve; nVIII: vestibulocochlear nerve; RL: rhombic lip.
(3.2 MB JPG)

Figure S2 Pontine Nuclei Development in Single and Compound *Robo1–2, Robo3*, *Slit1–2*, and *Phox2b* Mutant Mice (A–H) Ventral views of whole-mount brains at the level of pontine nuclei (PN) of E17.5 wild-type (WT) (A), *Slit1^−/−^* (B), *Slit2^−/−^* (C), *Robo1^−/−^* (D), *Robo2^−/−^* (E), *Robo1^−/−^;Robo2^−/−^* (F), *Slit1^−/−^;Slit2^−/−^* (G), and *Phox2b^−/−^* (H), hybridized with a *Barhl1* probe. No abnormalities are observed in single *Slit1–2* or *Robo1–2* mutants (B–E), whereas ectopic *Barhl1^+^* cells posterior to PN are similarly observed near the ventral midline of compound *Robo1;Robo2* (F) and *Slit1;Slit2* (G) mutant brains (arrows). Similar ectopias are observed in *Phox2b* mutants (arrows in [H]).
(I) lateral (to the left), and ventral (to the right) views of E15.5 *Robo3^−/−^* brain hybridized with the *Barhl1* probe. Note that the migrating stream of PN fail to undergo the final ventral migration but instead turn dorsally at the level of the trigeminal nerve (black arrow). In the ventral view, it is evident that PN fail to reach the ventral midline and form a nucleus (see also [16]). The arrows indicate the turning point of PN as indicated also by the arrow in (I).
(4 MB JPG)

Figure S3 Ectopic Migration of Subsets of PN in *Hoxa2* Mutant Mice (A–J) Analysis of PN migration defects by in situ hybridization of whole-mount hindbrains with *Pax6* at E13.0 (A and B, and E and F), *Robo3* at E14.0 (C and D, and G and H), and *Tag1* at E17.5 (I and J). Lateral (E–J) and ventral (A–D) views of *Hoxa2^+/−^* (A, E, I, C, and G) and *Hoxa2^−/−^* (B, F, J, D, and H) whole-mount brains. Note that ectopically migrating streams of PN are observed at all stages (arrows in [B, F, D, H, and J]), suggesting a random affection of PN migration induced by the loss of Hoxa2. (K and L) Coronal cryostat sections of E17.5 brains hybridized with *Barhl1*. The section level is indicated by the red line in the left diagram. The picture highlights the region depicted by the red box in the left diagram. The dashed lines show approximately the limits of the RTN and PGN, respectively. Note that a slight decrease of both the RTN and the PGN is observed in *Hoxa2^−/−^* brains. Arrows show the PGN and RTN.
nV: trigeminal nerve; nVIII: vestibulocochlear nerve.
(5.1 MB JPG)

## Abbreviations

AP: anteroposterior
ChIP: chromatin immunoprecipitation
Dcc: Deleted in colon cancer
DV: dorsoventral
E: embryonic day
ECN: external cuneate nucleus
FMN: facial motor nucleus
LRN: lateral reticular nucleus
P: postnatal day
PG: paralog group
PGN: pontine gray nucleus
PN: pontine neuron
r: rhombomere
RTN: reticulotegmental nucleus
